# Identifying a core symptom set triggering radiological and endoscopic investigations for suspected recurrent esophago-gastric cancer: a modified Delphi consensus process

**DOI:** 10.1093/dote/doac038

**Published:** 2022-07-21

**Authors:** Swathikan Chidambaram, Nikhil M Patel, Viknesh Sounderajah, Rita Alfieri, Luigi Bonavina, Edward Cheong, Andy Cockbain, Xavier Benoit D’Journo, Lorenzo Ferri, Ewen A Griffiths, Peter Grimminger, Caroline Gronnier, Christian Gutschow, Jakob Hedberg, Joonas H Kauppila, Sjoerd Lagarde, Donald Low, Philippe Nafteux, Grard Nieuwenhuijzen, Magnus Nilsson, Riccardo Rosati, Wolfgang Schroeder, B Mark Smithers, Mark I van Berge Henegouwen, Richard van Hillegesberg, David I Watson, Ravinder Vohra, Nick Maynard, Sheraz R Markar

**Affiliations:** Department of Surgery and Cancer, Imperial College London, London, UK; Department of Surgery and Cancer, Imperial College London, London, UK; Department of Surgery and Cancer, Imperial College London, London, UK; Department of Surgical Oncology, Veneto Institute of Oncology-IRCCS, Padova, Italy; Surgical Oncology of Digestive Tract, Veneto Institute of Oncology IOV-IRCCS Padua, Italy; Department of Upper Gastrointestinal Surgery, Norfolk and Norwich University Hospital, Norwich, UK; Department of Upper Gastrointestinal Surgery, Leeds Teaching Hospitals NHS Trust, Leeds, UK; Department of Thoracic Surgery, Aix-Marseille University, North Hospital, Marseille, France; Department of Thoracic Surgery and Upper Gastrointestinal Surgery, McGill University, Montréal, Canada; Department of Upper Gastrointestinal Surgery, Queen Elizabeth Hospital, University Hospitals Birmingham NHS Trust, Birmingham, UK; Institute of Cancer and Genomic Sciences, College of Medical and Dental Sciences, University of Birmingham, Birmingham, UK; Department of General Surgery, University of Mainz, Mainz, Germany; Digestive Surgery, CHU of Bordeaux, Bordeaux, France; Department of Surgery and Transplantation, University Hospital Zurich, Zurich, Switzerland; Section of Gastrointestinal Surgery, Department of Surgical Sciences, Uppsala University, Uppsala, Sweden; Department of Molecular Medicine and Surgery, Karolinska Institutet, Stockholm, Sweden; Department of Surgery, Oulu University Hospital, University of Oulu, Oulo, Finland; Department of Surgery, Erasmus University Medical Center, Rotterdam, the Netherlands; Department of Thoracic Surgery and Thoracic Oncology, Virginia Mason Medical Center, Seattle, WA, USA; Department of Thoracic Surgery, Katholieke Universiteit Leuven, Leuven, Belgium; Department of Surgery, Catharina Hospital, Eindhoven, the Netherlands; Division of Surgery, Department of Clinical Science, Intervention and Technology, Karolinska Institutet, Stockholm, Sweden; Department of Upper Abdominal Diseases, Karolinska University Hospital, Stockholm, Sweden; Department of Surgery, San Raffaele Hospital, Milan, Italy; Department of General, Visceral, Cancer and Transplantation Surgery, University of Cologne, Cologne, Germany; Upper GI and Soft Tissue Unit, Academy of Surgery, Princess Alexandra Hospital, Faculty of Medicine, University of Queensland, Brisbane, Australia; Department of Surgery, Amsterdam University Medical Centers, Amsterdam, the Netherlands; Department of Surgery, University Medical Center Utrecht, Utrecht, the Netherlands; Discipline of Surgery, College of Medicine and Public Health, Flinders University, Adelaide, Australia; Department of Surgery, Flinders Medical Center, Adelaide, Australia; Department of Esophagogastric Surgery, Nottingham University Hospitals NHS Trust, Nottingham, UK; Department of Upper GI Surgery, Oxford University Hospitals NHS Foundation Trust, Oxford, UK; Department of Surgery and Cancer, Imperial College London, London, UK; Department of Molecular Medicine and Surgery, Karolinska Institutet, Stockholm, Sweden; Department of Upper GI Surgery, Oxford University Hospitals NHS Foundation Trust, Oxford, UK

**Keywords:** esophageal cancer, gastric cancer, surveillance, symptom, endoscopic

## Abstract

Background: There is currently a lack of evidence-based guidelines regarding surveillance for recurrence after esophageal and gastric (OG) cancer surgical resection, and which symptoms should prompt endoscopic or radiological investigations for recurrence. The aim of this study was to develop a core symptom set using a modified Delphi consensus process that should guide clinicians to carry out investigations to look for suspected recurrent OG cancer in previously asymptomatic patients. Methods: A web-based survey of 42 questions was sent to surgeons performing OG cancer resections at high volume centers. The first section evaluated the structure of follow-up and the second, determinants of follow-up. Two rounds of a modified Delphi consensus process and a further consensus workshop were used to determine symptoms warranting further investigations. Symptoms with a 75% consensus agreement as suggestive of recurrent cancer were included in the core symptom set. Results: 27 surgeons completed the questionnaires. A total of 70.3% of centers reported standardized surveillance protocols, whereas 3.7% of surgeons did not undertake any surveillance in asymptomatic patients after OG cancer resection. In asymptomatic patients, 40.1% and 25.9% of centers performed routine imaging and endoscopy, respectively. The core set that reached consensus, consisted of eight symptoms that warranted further investigations included; dysphagia to solid food, dysphagia to liquids, vomiting, abdominal pain, chest pain, regurgitation of foods, unexpected weight loss and progressive hoarseness of voice. Conclusion: There is global variation in monitoring patients after OG cancer resection. Eight symptoms were identified by the consensus process as important in prompting radiological or endoscopic investigation for suspected recurrent malignancy. Further randomized controlled trials are necessary to link surveillance strategies to survival outcomes and evaluate prognostic value.

## INTRODUCTION

Esophago-gastric (OG) cancers are associated with a range of symptoms, including progressive dysphagia and weight loss.^1^ These symptoms may also be associated with recurrent disease after initial curative treatment, which typically involves a combination of surgery, and chemotherapy alone or combined chemoradiotherapy.^2^ Symptoms such as dysphagia or weight loss may trigger endoscopic and radiological investigations on a suspected cancer pathway, which is designed to diagnose OG malignancies in a timely manner.^3^ However, in the United Kingdom, the National Institute of Clinical Excellence (NICE) does not have any standardized recommendations for follow-up after curative treatment for OG cancer in the previously asymptomatic patient.^4^^,^^5^ Furthermore, follow-up protocols vary considerably on a global scale.^11–13^ For example, in Japan, high intensity follow-up involving both computed tomography (CT) and endoscopy is advocated in many high-volume OG centers, and in some centers in the United States three monthly CT scans and blood tests are recommended for the first 3 years after curative treatment.^6^^,^^13^

Unlike other GI cancers, there is an international lack of consensus concerning standardized surveillance protocols following curative treatment of OG cancer.^7^ Previous observational studies have evaluated different surveillance protocols consisting of clinical examination, imaging and endoscopy; however, they are usually underpowered to show significant differences.^8^ Furthermore, follow-up regimens vary considerably between OG centers. In fact, we have previously captured this variation in clinical practice and physician attitudes towards surveillance within the UK’s centers.^9^ As such, patients may have developed advanced disease by the time they report new and unexpected symptoms suggestive of recurrent cancer. For example, symptoms such as dysphagia may suggest post-operative complications such as a benign anastomotic stricture, or recurrent cancer, especially if experienced later on in the timeline since surgical intervention.^10^ It is important to recognize the presentation of specific new onset symptoms in a previously asymptomatic patient require further investigations for recurrence, so that clinicians can offer appropriate treatment options early enough to optimize survival outcomes. In this study, we aim to identify a core set of new-onset symptoms that should trigger radiological or endoscopic investigations following OG cancer resection through an electronic Delphi (modified Delphi) consensus process.

### Methods

This study is the culmination of a larger evidence generation process on surveillance after resection of OG cancer.^9^^,^^11^ The Delphi methodology was used based on previous similar evidence generation processes within the field.^12^^,^^13^ This modified Delphi consensus was carried out in three stages using Qualtrics, an online survey platform (Qualtrics XM, USA). Access to Qualtrics was provided by Imperial College London. For each stage, a link to an online survey was sent via e-mail to selected consultant/attending-level OG surgeons working in high-volume centers in the UK, Europe, North America and Australia. Both centers and respective surgeons were selected based on the volume of OG cancer resections performed on average each year. Initially, a scoping survey was performed that sought to delineate factors such as case volume per year, variation in frequency and modality of follow-up investigations after surgery for OG cancer and clinical factors such as tumor histology and post-operative complications in triggering endoscopic or radiological follow-up investigations.

The second stage of the consensus included two rounds which were pre-determined at study conception. A set of 28 symptoms was sent to all participating surgeons to start to identify the core symptom set triggering endoscopic or radiological investigations for suspected cancer recurrence. These symptoms were selected based on the Lasting Symptoms After Esophageal Resection (LASER) study where they were the most prevalent seen by long-term survivors following surgery.^14^ Surgeons were therefore asked to rate, in their opinion, the level of importance of each symptom at triggering radiological or endoscopic investigation to detect cancer recurrence, using a 5-point Likert scale (1 = not at all important, 5 = extremely important). After the first round, any symptoms with a 75% or higher overall consensus were accepted if deemed as important in triggering endoscopic or radiological investigations. The value of 75% was used as consensus threshold based on a systematic review by Diamond *et al.* on methodology in reporting Delphi studies.^15^ All remaining symptoms were carried forward into the second round of the Delphi where this process was repeated to generate the final core symptom set. Feedback was anonymized between rounds, and the data from each round was collated and presented to the invited surgeons. Both rounds of modified Delphi were user tested prior to being sent. Finally, a third virtual consensus workshop was organized to discuss the addition of other symptoms that were not initially covered by the questionnaire but proposed in the open-ended sections of the first two rounds. All data are expressed as percentages where frequency or proportions are reported. The complete questionnaire and the list of participating centers are provided as a supplementary file (Supplementary file 1).

## RESULTS

### Characteristics of survey responders

Twenty-seven surgeons completed the questionnaire. Of the survey responders, all were consultants or attending surgeons. Six were from the UK; two from Australia; three from North America; and the remaining sixteen from European centers. All responses came from centers that performed at least 20 esophageal resections per year, with over 59.3% of centers carrying out more than 60 resections. Centers generally carried out fewer gastric resections with 88.9% of centers performing fewer than 60 gastric resections. Seven centers performed more than 100 esophageal resections, although none performed over 100 gastric procedures in a year. Furthermore, 74.1% of centers had fewer than five surgeons, while the remainder had 5–10 surgeons undertaking these resections.

### Pattern of surveillance protocols

All patients were followed up by a member of the surgical team in the outpatient clinic setting. For both esophageal and gastric cancers, 96.3% of surgical departments arranged routine follow-up, while the remainder followed their patients based on specific clinician or patient related factors. After esophagectomy or gastrectomy, 70.3% of centers reported standardized surveillance protocols for all patients, while 29.6% tailored it to patient and physician preferences. One center did not undertake surveillance of asymptomatic patients. The average time taken to the first outpatient follow-up appointment after discharge varied from 2 weeks to more than 6 weeks, with 75% of centers, seeing patients within 4 weeks of surgery, and two centers at more than 6 weeks post-surgery.

**Fig. 1:**
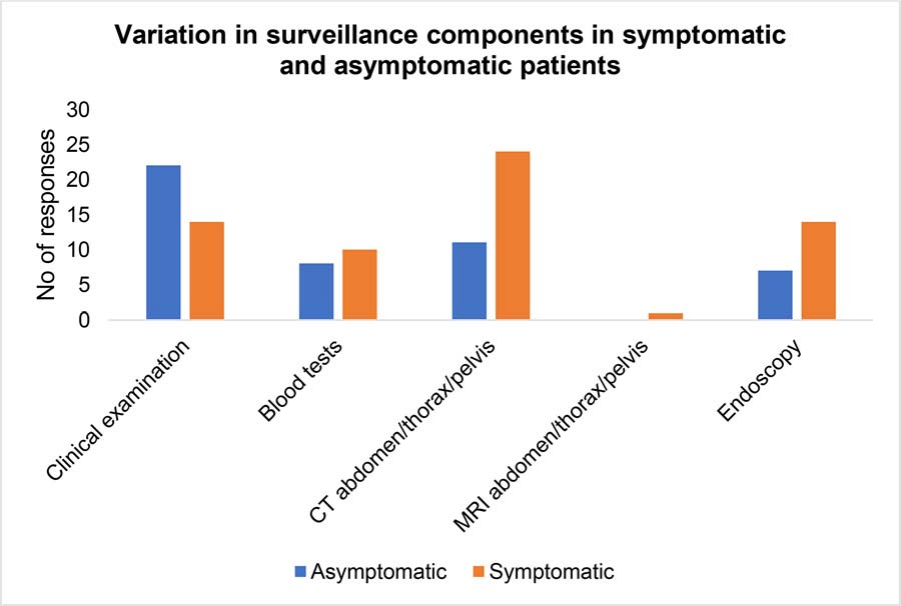
Variation in surveillance components in symptomatic and asymptomatic patients.

The components of follow-up varied by center (Fig. 1). In asymptomatic patients, 81.4% performed clinical examination of the chest and abdomen, while 29.6% also carried out blood tests such as basic blood panel and tumor markers. Furthermore, 40.7% arranged routine CT scan of the thorax, abdomen and pelvis. In 25.9% of centers, patients also underwent planned esophagogastroduodenoscopy during follow-up. In contrast, 51.8% only perform clinical examination on symptomatic patients. A total of 37% of surgeons also arranged for blood tests. In symptomatic patients, 88.8% surgeons reported they arrange for CT scan of the thorax, abdomen, and pelvis, while 51.8% would also opt to carry out endoscopy. Lastly, 31% routinely discuss patients in a multi-disciplinary team (MDT) setting if requiring endoscopic or radiological investigation for possible cancer recurrence. In 23.1%, this is done on a case-by-case basis. Of note 7.7% centers never discuss patients in an MDT even if cancer recurrence was suspected on endoscopy or cross-sectional imaging.

### Factors prompting further investigations in surveillance

**Fig. 2:**
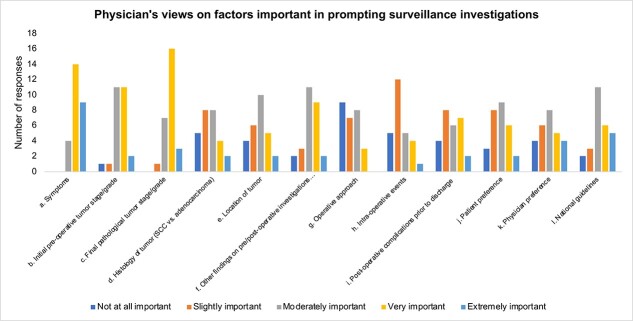
Factors to prompt surveillance investigation for possible cancer recurrence.

New symptoms were the most important factor initiating investigations during follow-up (Fig. 2). The majority of centers (85.2%) reported this as either a very important or extremely important factor. This was closely followed by pathological tumor stage/grade, which was rated as important by 70.4% of surgeons. Half of the surgeons also rated initial pre-operative tumor stage/grade as important in influencing post-operative surveillance, while 41% mentioned findings on investigations such as blood tests, imaging and endoscopy as a crucial determinant. This was closely followed by patient preference, physician preference and national guidelines as important factors in initiating further surveillance, with 37%, 33.3% and 40.7% of surgeons rating them as important. Other tumor-level factors such as location and post-operative histology were not considered important (22.2% and 25.9%, respectively). Operative factors such as surgical technique (open/laparoscopic/robotic) or intra-operative events were not considered as important, with 59.2% and 62.9% of responding surgeons rating each as either not at all or only slightly important respectively. Post-operative events prior to discharge and first clinical visit did not rank highly either, with only 33.3% rating it as important.

### New-onset symptoms prompting further investigations in surveillance

In round 1 of the Delphi process, only three symptoms reached consensus as important new onset symptoms in a previously asymptomatic patient to initiate further investigations during surveillance. These included dysphagia to solid food; dysphagia to liquids; and vomiting. In contrast, three symptoms were deemed as unimportant, including intermittent voice problems, pain from scars on the chest, and pain from scars on the abdomen. These six symptoms were not carried onto the second round of the Delphi process (Table 1). From the second round, three further symptoms reached consensus as important in association with recurrence, including abdominal pain (88.4%), chest pain (84.6%), and regurgitation of foods (84.6%) (Table 2). Four symptoms were not deemed as important after round 2, which included sweating after eating (19.2%), dizziness after eating (19.2%), loose bowel motions or diarrhea after eating (15.4%) and low mood (15.4%). In the third online consensus workshop, two symptoms, namely unexpected weight loss and progressive hoarseness of voice, were added after reaching 100% consensus amongst the attendees. The remainder of the symptoms failed to reach consensus after two rounds of the Delphi process (Table 2).

**Table 1:** Consensus of core symptoms set for round 1 of modified Delphi process

**Symptom**	**Accepted on round 1 (% consensus)**	**Dropped on round 1 (% consensus)**
Difficulty getting food down	100	
Difficulty getting liquids down	100	
Vomiting	85.18	
Intermittent voice problems		21.42
Pain from scars on chest		25.93
Pain from scars on abdomen		22.22
Abnormal sensation in toes and fingers		3.70
Dental problems		7.41

**Table 2:** Consensus of core symptoms set for rounds 2 and 3 of the modified Delphi process. Data are not shown for symptoms accepted on round 3, as the process was not carried out

**Symptom**	**Accepted on round 2 (% consensus)**	**Dropped on round 2 (% consensus)**	**No consensus achieved (% consensus)**	**Proposed and accepted on round 3**
Chest pain	40 → 84.62			
Abdominal pain	60 → 88.46			
Regurgitation of foods	72 → 84.62			
Nausea			56 → 57.69	
Early feeling of fullness after eating			48 → 53.85	
Heart palpitations after eating			28 → 23.08	
Sweating after eating		28 → 19.23		
Dizziness after eating		28 → 19.23		
Bloating or cramping after eating			36 → 42.3	
Loose bowel movements or diarrhea after eating		28 → 15.38		
Heartburn/acid/bile (sour/bitter tasting) regurgitation			36 → 46.15	
Waking up during the night because of choking			46 → 46.15	
Persistent cough			52 → 73.08	
Stools that float and are difficult to flush			28 → 23.08	
Diarrhea (>3 times per day) unrelated to eating			36 → 26.92	
Lack of appetite			56 → 65.39	
Tiredness			48 → 57.69	
Low mood		24 → 15.39		
Reduced energy/activity tolerance			48 → 38.46	
Hiccups			52 → 76.93	
Weight loss				N/A
Progressive hoarseness of voice				N/A

## DISCUSSION

This study highlights the large degree of global heterogeneity between centers relating to post-operative surveillance after esophageal or gastric resection for cancer. In addition to this, it reflects the lack of standardized national protocols for surveillance of patients after surgery for esophageal and gastric cancers in high volume international centers. Furthermore, it highlights a discrepancy between surgeons with respect to the most important factors influencing how surveillance is undertaken. As expected, investigations were not routine. The most pertinent factor was whether the patient was symptomatic or not. Of the various possible symptoms given, our respondents indicated that the new onset of eight of these in a patient who has previously undergone OG resection for cancer and has been asymptomatic, were suggestive of possible disease recurrence and should stimulate further evaluation with endoscopic or radiological investigations. The selected symptoms were abdominal pain, chest pain, dysphagia to solid food, dysphagia to liquids, regurgitation of foods, vomiting, unexpected weight loss and progressive hoarseness of voice. In asymptomatic patients, the components of surveillance varied between centers, reflecting the need for further research in this area.

These results are consistent with a previous study in the UK that identified a similar variation in follow-up periods, frequencies, components, investigations arranged for patients and factors that should be taken into consideration.^9^ Compared with that study, the disparity in surveillance strategies was wider when comparing with centers outside the UK. This reflects practice variations and the little national or international guidance on how patients should be monitored after OG cancer surgery, and thus strongly identifies an area for further work. Nevertheless, there was overwhelming agreement that clinical presentation was the most important factor to initiate further imaging or endoscopy with eight of the symptoms reaching consensus for inclusion in the core symptom set, and 15 which did not achieve consensus despite overall agreement to be of importance to trigger further investigation for recurrence. This, again, shows a degree of subjectivity in organizing follow-up, even with the assessment of patient reported symptoms.

It is interesting that patient preference was ranked as a crucial factor by only 35.9% of respondents. This contradicts the increasing emphasis of patient-reported outcome measures in all other parts of cancer pathways globally. This may be to avoid any potential anxiety resulting in investigations for otherwise asymptomatic patients. However, unnecessary anxiety can also result from worries about recurrence, so negative CT scans and endoscopy may reassure patients as well. There is compelling evidence outlining patients showed a strong preference for routine surveillance after esophagectomy, with one study of 45 patients who underwent esophagectomy with curative intent reporting that 67% of these surveyed patients prefer imaging even if this does not improve survival outcomes.^16^ Furthermore, an equal number of respondents indicated national guidelines to be an important but neutral factor in triggering investigations amongst asymptomatic patients. This may be due to the lack of standardized guidelines on how surveillance should be structured. Currently, many countries lack national guidelines on how to carry out surveillance in patients who have undergone treatment for OG cancers (surgical and/or non-surgical), primarily due to lack of evidence. Hence, there is a strong need for further research to study the structure of surveillance protocols in this cohort of patients in the advent of newer systemic therapies.

The LASER study identified key symptoms that correlated with poor quality of life (QOL).^14^ Based on 876 patients from over 20 centers, the three symptoms from the LASER questionnaire that were significantly associated with poor HRQOL as measured by the validated EORTC QLQ-C30 and QLQ-OG25 tools were: low mood, pain from scars on chest, and reduced energy or activity tolerance. Our current questionnaire identified the following symptoms to require further evaluation given the potential association with recurrence: abdominal pain, chest pain, dysphagia to solid food, dysphagia to liquid food, regurgitation of foods, vomiting, weight loss and progressive hoarseness of voice.

Furthermore, some of the symptoms may be related to underlying reflux or dumping syndrome that are actually frequent functional long-term sequelae of OG surgery. This suggests a potential divergence between what symptoms are acceptable to patients and physicians,; the distinction between ‘functional’ and alarming ‘red flag’ symptoms; and that symptoms that would concern physicians for recurrence usually do not trouble patients as much, and vice versa. While recurrence of cancer may reduce the QOL, the direct symptoms which may suggest recurrence do not necessarily tally with PROMs of poor QOL. This further emphasizes the need to combine both patient and physician views when arranging further follow-up investigations, both for detecting recurrence and to improve patient’s QOL.

The majority of surgeons that took part in our study reported that they seldom or never routinely discussed patients in an MDT forum if they were suspected to have recurrence based on clinical presentation and imaging. Previously, more treatment options are available for managing recurrent OG cancers with the advent of novel surgical and oncological regimens.^17^ Even if curative care is not possible, early introduction of palliative specialists can give better survival as well as QOL making MDTs crucial in this setting.^18^ Of all the symptoms, it is unsurprising that difficulty getting food and liquids down, as well as vomiting and regurgitation of foods are included in the consensus as these obstructive symptoms are common in the index presentation of OG cancer. Weight loss was also a symptom highlighted by this modified Delphi that is an important additional symptom suggestive of cancer recurrence, given that this is particular symptom is associated with all cancers, both initial presentation and recurrent disease.^19^

The study is important in characterizing how clinicians organize follow-up for patients who undergo surgical intervention. Given that the risk of residual or recurrent cancer in this cohort remains unquantified, patients may benefit from more regular surveillance that could ideally include cross-sectional imaging and endoscopy. Our study provides an indication of the factors that should be considered in designing such protocols. Further work should be aimed at understanding physician attitudes towards surveillance in cohorts that have not undergone surgery and only chemoradiotherapy, and subsequently map any differences in strategies used.

A major strength of our study is that it involved surgeons from high-volume tertiary centers globally, including Australia, Europe and North America. Through this, it captured physician attitudes and practice variations in how surveillance is carried out at both a national and international level. The main limitation of our study is that it is entirely clinician focused, and consists only of senior surgeons. The survey was completed by a single surgeon who responds to all the questions. The questions do not distinguish separately between esophageal and gastric cancers, but instead group them together. Thus, it could be argued that there may be certain symptoms prompting investigations after esophagectomy and slightly different symptoms in the case of post-gastrectomy patients. There may also be other rarer symptoms such as shortness of breath or bony pain, which were not considered by the panel but may be seen with cancer recurrence. Further work could involve investigating the relationship between tumor biology and surveillance protocols, while looking at disease-free survival as the potential outcome measure. The results can be useful in generating updated clinical guidelines for the surveillance of esophageal and gastric cancers.

In conclusion, the paucity of evidence-based guidelines for post-operative surveillance after esophago-gastric resections has led to considerable variation on how patients are monitored for recurrence, especially if they are asymptomatic. Despite significant variations between experts on the majority of symptoms, a consensus on eight symptoms that should trigger further investigations in previously asymptomatic patients was achieved in this study. Other important patient and disease-related factors should be considered in how surveillance is carried out, specifically, what investigations it should comprise of and how frequently they should be performed. Further prospective, large-scale national trials are required to standardize how monitoring for recurrence should be undertaken in symptomatic and asymptomatic patients, and link these to both survival outcomes and patient-related outcomes on quality of life. The eight-symptom tool generated here should further be validated within existing cohorts to test if it is possible to identify patients at risk of recurrence based upon symptoms alone.

## Supplementary Material

Supplementary_table_1_doac038

## Notes

Synopsis: There is a lack of evidence-based guidelines regarding surveillance after surgical resection of esophago-gastric cancer. This modified Delphi consensus identified eight core symptoms identified as important in guiding the surgeon to carry out radiological and endoscopic investigations to search for disease recurrence after surgical resection.
