# Supplementary material for: Identifying a core symptom set triggering radiological and endoscopic investigations for suspected recurrent esophago-gastric cancer: a modified Delphi consensus process
Source: Dis Esophagus. 2022 Jul 21;36(1):doac038. doi: 10.1093/dote/doac038 (PMC9817822; doi:10.1093/dote/doac038)
Supplement: Supplementary_table_1_doac038 [file supplementary_table_1_doac038.docx]

Radiological surveillance

Start of Block: Demographics

Q1 1. What is the name of the person completing this survey

________________________________________________________________

Q2 2. What is the name of your hospital?

________________________________________________________________

Q3 3. What country is your center based in?

________________________________________________________________

Q4 4. Which continent are you based in?

- a. Asia (1)
- b. Australia (2)
- c. Europe (3)
- d. North America (4)
- e. South America (5)

Q5 5. What is the role of the person completing this survey?

- a. Surgeon (1)
- b. Oncologist (2)
- c. Clinical nurse specialist (3)
- d. Other auxiliary healthcare professional (4)

Q6 6. How many esophageal resections does your centre perform annually?

- a. 0 to 20 (1)
- b. 21 to 40 (2)
- c. 41 to 60 (3)
- d. 61 to 80 (4)
- e. 81 to 100 (5)
- f. >100 (6)

Q7 7. How many gastric resections does your centre perform annually?

- Click to write Choice 1 (1)
- a. 0 to 20 (2)
- b. 21 to 40 (3)
- c. 41 to 60 (4)
- d. 61 to 80 (5)
- e. 81 to 100 (6)
- f. >100 (7)

Q8 8. How many consultants undertake oesophageal and gastric cancer resections at your centre?

- a. (1)
- b. 5 to 10 (2)
- c. >10 (3)

End of Block: Demographics

Start of Block: Block 1

Q9 1. Does your unit routinely follow up patients in the outpatient setting after esophageal resection for cancer?

- a. Yes (1)
- b. No (2)
- c. Varies depending on physician and patient preferences/factors (3)

Q10 2. Does your unit routinely follow up patients in the outpatient setting after gastric resection for cancer?

- a. Yes (1)
- b. No (2)
- c. Varies depending on physician and patient preferences/factors (3)

Q11 3. What is your follow up protocol after oesophagectomy for cancer?

- a. Standardised for all patients (1)
- b. Tailored to patient and physician preferences/factors (2)
- c. Surveillance of asymptomatic patients is not undertaken (3)
- d. No surveillance undertaken at all (4)

Q12 4. What is your follow up protocol after gastrectomy for cancer?

- a. Standardised for all patients (1)
- b. Tailored to patient and physician preferences/factors (2)
- c. Surveillance of asymptomatic patients is not undertaken (3)
- d. No surveillance undertaken at all (4)

Q13 5. What is the average time taken for first clinic follow-up from day of discharge?

- a. 0-2 weeks (1)
- b. 2-4 weeks (2)
- c. 4-6 weeks (3)
- d. >6 weeks (4)

End of Block: Block 1

Start of Block: Investigations at surveillance

Q15 1. Are any of the following investigations routinely undertaken at follow-up?

- a. Clinical examination (1)
- b. Blood tests (2)
- c. CT abdomen/thorax/pelvis (3)
- d. MRI abdomen/thorax/pelvis (4)
- e. Endoscopy (5)

Q16 Are any of the following investigations undertaken at follow-up for symptomatic patients?

- a. Clinical examination (1)
- b. Blood tests (2)
- c. CT abdomen/thorax/pelvis (3)
- d. MRI abdomen/thorax/pelvis (4)
- e. Endoscopy (5)

Q17 3. How important are the following symptoms to you in prompting endoscopic or radiological investigation for possible cancer recurrence?

|  | Not at all important (1) | Slightly important (2) | Moderately important (3) | Very important (4) | Extremely important (5) |
| --- | --- | --- | --- | --- | --- |
| a.a. Chest pain (1) |  |  |  |  |  |
| b. Abdominal pain (7) |  |  |  |  |  |
| c. Pain from scars on chest (8) |  |  |  |  |  |
| d. Pain from scars on abdomen (9) |  |  |  |  |  |
| e. Difficulty getting food down (10) |  |  |  |  |  |
| f. Difficulty getting liquids down (11) |  |  |  |  |  |
| g. Regurgitation of food (12) |  |  |  |  |  |
| h. Nausea (13) |  |  |  |  |  |
| i. Vomiting (14) |  |  |  |  |  |
| j. Early feeling of fullness after eating (15) |  |  |  |  |  |
| k. Heart palpitations after eating (16) |  |  |  |  |  |
| l. Sweating after eating (17) |  |  |  |  |  |
| m. Dizziness after eating (18) |  |  |  |  |  |
| n. Bloating or cramping after eating (19) |  |  |  |  |  |
| o. Loose bowel motions or diarrhea after eating (20) |  |  |  |  |  |
| p. Heartburn/acid/bile (sour/bitter tasting) regurgitation (21) |  |  |  |  |  |
| q. Waking up during the night because of choking (22) |  |  |  |  |  |
| r. Persistent cough (23) |  |  |  |  |  |
| s. Stools that float and are difficult to flush (24) |  |  |  |  |  |
| t. Diarrhea (>3 times per day) unrelated to eating (25) |  |  |  |  |  |
| u. Lack of appetite (26) |  |  |  |  |  |
| v. Tiredness (27) |  |  |  |  |  |
| w. Low mood (28) |  |  |  |  |  |
| x. Reduced energy/activity tolerance (29) |  |  |  |  |  |
| y. Voice problems (30) |  |  |  |  |  |
| z. Abnormal sensation in fingers and toes (31) |  |  |  |  |  |
| aa. Dental problems (32) |  |  |  |  |  |
| bb. Hiccups (33) |  |  |  |  |  |

Q18 4. How important are the following factors to you in prompting endoscopic or radiological investigation for possible cancer recurrence?

|  | Not at all important (1) | Slightly important (2) | Moderately important (3) | Very important (4) | Extremely important (5) |
| --- | --- | --- | --- | --- | --- |
| a. Symptoms (1) |  |  |  |  |  |
| b. Initial pre-operative tumor stage/grade (2) |  |  |  |  |  |
| c. Final pathological tumor stage/grade (3) |  |  |  |  |  |
| d. Histology of tumor (SCC vs. adenocarcinoma) (4) |  |  |  |  |  |
| e. Location of tumor (5) |  |  |  |  |  |
| f. Other findings on pre/post-operative investigations (bloods/imaging/endoscopy) (6) |  |  |  |  |  |
| g. Operative approach (7) |  |  |  |  |  |
| h. Intra-operative events (8) |  |  |  |  |  |
| i. Post-operative complications prior to discharge and first clinical follow-up (9) |  |  |  |  |  |
| j. Patient preference (10) |  |  |  |  |  |
| k. Physician preference (11) |  |  |  |  |  |
| l. National guidelines (12) |  |  |  |  |  |

Q19 5. Are there any additional symptoms not listed above which would prompt you to carry out endoscopic or radiological investigations for suspected recurrence?

________________________________________________________________

Q20 6. Are patients discussed in a MDT setting if requiring endoscopic or radiological investigation for possible cancer recurrence?

- a. Yes, always (1)
- b. Sometimes, on a case-by-case basis (2)
- c. Not routinely (3)
- d. Never (4)

End of Block: Investigations at surveillance

Start of Block: Block 3

Surveillance Delphi (round 2)

Start of Block: Symptoms

What is the name of the person completing this survey

________________________________________________________________

**40%** of respondents indicated that "**chest pain**" is a moderately-extremely important symptom. 
 
How important is this symptom to you in triggering further radiological/endoscopic investigations?

- Not at all important (1)
- Slightly important (2)
- Moderately important (3)
- Very important (4)
- Extremely important (5)

**60%** of respondents indicated that "**abdominal pain**" is a moderately-extremely important symptom. 
 
How important is this symptom to you in triggering further radiological/endoscopic investigations?

- Not at all important (1)
- Slightly important (2)
- Moderately important (3)
- Very important (4)
- Extremely important (5)

**72%** of respondents indicated that "**regurgitation of foods**" is a moderately-extremely important symptom. 
 
How important is this symptom to you in triggering further radiological/endoscopic investigations?

- Not at all important (1)
- Slightly important (2)
- Moderately important (3)
- Very important (4)
- Extremely important (5)

**56%** of respondents indicated that "**nausea**" is a moderately-extremely important symptom. 
 
How important is this symptom to you in triggering further radiological/endoscopic investigations?

- Not at all important (1)
- Slightly important (2)
- Moderately important (3)
- Very important (4)
- Extremely important (5)

**48%** of respondents indicated that "**Early feeling of fullness after eating**" is a moderately-extremely important symptom. 
 
How important is this symptom to you in triggering further radiological/endoscopic investigations?

- Not at all important (1)
- Slightly important (2)
- Moderately important (3)
- Very important (4)
- Extremely important (5)

**28%** of respondents indicated that "**Heart palpitations after eating**" is a moderately-extremely important symptom. 
 
How important is this symptom to you in triggering further radiological/endoscopic investigations?

- Not at all important (1)
- Slightly important (2)
- Moderately important (3)
- Very important (4)
- Extremely important (5)

**28%** of respondents indicated that "**Sweating after eating** " is a moderately-extremely important symptom. 
 
How important is this symptom to you in triggering further radiological/endoscopic investigations?

- Not at all important (1)
- Slightly important (2)
- Moderately important (3)
- Very important (4)
- Extremely important (5)

**28%** of respondents indicated that "**Dizziness** **after eating** " is a moderately-extremely important symptom. 
 
How important is this symptom to you in triggering further radiological/endoscopic investigations?

- Not at all important (1)
- Slightly important (2)
- Moderately important (3)
- Very important (4)
- Extremely important (5)

**36%** of respondents indicated that "**Bloating or cramping after eating** " is a moderately-extremely important symptom. 
 
How important is this symptom to you in triggering further radiological/endoscopic investigations?

- Not at all important (1)
- Slightly important (2)
- Moderately important (3)
- Very important (4)
- Extremely important (5)

**28%** of respondents indicated that "**Loose bowel motions or diarrhea after eating**" is a moderately-extremely important symptom. 
 
How important is this symptom to you in triggering further radiological/endoscopic investigations?

- Not at all important (1)
- Slightly important (2)
- Moderately important (3)
- Very important (4)
- Extremely important (5)

**36%** of respondents indicated that "**Heartburn/acid/bile (sour/bitter tasting) regurgitation**" is a moderately-extremely important symptom. 
 
How important is this symptom to you in triggering further radiological/endoscopic investigations?

- Not at all important (1)
- Slightly important (2)
- Moderately important (3)
- Very important (4)
- Extremely important (5)

**46%** of respondents indicated that "**Waking up during the night because of choking**" is a moderately-extremely important symptom. 
 
How important is this symptom to you in triggering further radiological/endoscopic investigations?

- Not at all important (1)
- Slightly important (2)
- Moderately important (3)
- Very important (4)
- Extremely important (5)

**52%** of respondents indicated that "**Persistent cough**" is a moderately-extremely important symptom. 
 
How important is this symptom to you in triggering further radiological/endoscopic investigations?

- Not at all important (1)
- Slightly important (2)
- Moderately important (3)
- Very important (4)
- Extremely important (5)

**28%** of respondents indicated that "**Stools that float and are difficult to flush**" is a moderately-extremely important symptom. 
 
How important is this symptom to you in triggering further radiological/endoscopic investigations?

- Not at all important (1)
- Slightly important (2)
- Moderately important (3)
- Very important (4)
- Extremely important (5)

**36%** of respondents indicated that "**Diarrhea (>3 times per day) unrelated to eating**" is a moderately-extremely important symptom. 
 
How important is this symptom to you in triggering further radiological/endoscopic investigations?

- Not at all important (1)
- Slightly important (2)
- Moderately important (3)
- Very important (4)
- Extremely important (5)

Q39 **56%** of respondents indicated that "**Lack of appetite**" is a moderately-extremely important symptom. 
 
How important is this symptom to you in triggering further radiological/endoscopic investigations?

- Not at all important (1)
- Slightly important (2)
- Moderately important (3)
- Very important (4)
- Extremely important (5)

**48%** of respondents indicated that "**Tiredness**" is a moderately-extremely important symptom. 
 
How important is this symptom to you in triggering further radiological/endoscopic investigations?

- Not at all important (1)
- Slightly important (2)
- Moderately important (3)
- Very important (4)
- Extremely important (5)

**24%** of respondents indicated that "**Low mood**" is a moderately-extremely important symptom. 
 
How important is this symptom to you in triggering further radiological/endoscopic investigations?

- Not at all important (1)
- Slightly important (2)
- Moderately important (3)
- Very important (4)
- Extremely important (5)

**48%** of respondents indicated that "**Reduced energy/activity tolerance**" is a moderately-extremely important symptom. 
 
How important is this symptom to you in triggering further radiological/endoscopic investigations?

- Not at all important (1)
- Slightly important (2)
- Moderately important (3)
- Very important (4)
- Extremely important (5)

**52%** of respondents indicated that "**Hiccups**" is a moderately-extremely important symptom. 
 
How important is this symptom to you in triggering further radiological/endoscopic investigations?

- Not at all important (1)
- Slightly important (2)
- Moderately important (3)
- Very important (4)
- Extremely important (5)

End of Block: Symptoms
